# Application of non-invasive prenatal testing in screening chromosomal aberrations in pregnancies with different nuchal translucency cutoffs

**DOI:** 10.1186/s13039-023-00661-1

**Published:** 2023-10-28

**Authors:** Yong Xu, Siqi Hu, Liyuan Chen, Ying Hao, Hu Zhang, Zhiyong Xu, Weiqing Wu, Liyanyan Deng

**Affiliations:** 1 Medical Genetics Center, Affiliated Shenzhen Maternity & Child Healthcare Hospital, Southern Medical University, Shenzhen, Guangdong China; 2 Peripheral Vascular Ward (Cardiac Surgery Ward 1), Fuwai Hospital Chinese Academy of Medical Sciences, Shenzhen, Guangdong China

**Keywords:** Non-invasive prenatal testing (NIPT), Nuchal translucency (NT), Chromosomal diagnostic testing, Chromosomal aberrations, Residual risk

## Abstract

**Objective:**

To investigate the efficiency of non-invasive prenatal testing (NIPT) in cases with different cutoffs of nuchal translucency (NT).

**Methods:**

The study retrospectively analyses pregnancies with NT ≥ 2.5 mm who underwent NIPT. Results of NT, NIPT, chromosomal diagnostic and pregnancy outcomes were collected.

**Results:**

Study group was composed of 1470 single pregnancies, including 864 with NT 2.5–2.9 mm, 350 with NT 3.0–3.4 mm and 256 with NT ≥ 3.5 mm. Non-significant differences were found in the positive predictive value (PPV) of NIPT between different cutoffs of NT. There was one false positive case with NT 4.3 mm, screening for 47,XYY in NIPT showed normal in diagnostic testing. For cases with normal NIPT results, the residual risk is 1:20 (5%, 95%CI: 0.1–10.1%) in fetuses with NT 3.0–3.4 mm and 1:15 (6.5%, 95%CI: 1.4%-11.5%) in fetuses with NT ≥ 3.5 mm. These false negative cases included one trisomy 21, seven pathogenic CNVs, one uniparental disomy and one single gene disorders.

**Conclusion:**

Our findings demonstrated that the PPV of NIPT for screening chromosomal aberrations were similarly in different NT cutoffs, while false positive case does exist. After normal in NIPT, risk for chromosomal aberrations remained, especially pathogenic CNV and even common trisomy. Therefore, prenatal diagnosis was recommended and CMA was suggested to apply in pregnancies with NT ≥ 3.0 mm.

## Introduction

Nuchal translucency (NT) measurement is widely used as a marker for fetal chromosomal abnormalities and congenital heart defects. NT alone enables identification of 70% of fetuses with trisomy 21 with a false positive rate of 5% [1]. Non-invasive prenatal testing (NIPT) has been widely used to screen fetal aneuploidies with the analysis of cell-free DNA (cfDNA) fragments in the maternal circulation. It was well established that NIPT is a superior screen for the common autosomal trisomies 13, 18 and 21 [1–3]. Although the occurring rate is low, discordant findings between NIPT and chromosomal diagnostic testing are remained globally [4–6]. In this circumstance, there is debate over the utility of NT in the era of NIPT. Some experts support the combined use of NT and NIPT, stating that NT may identify pregnancies at risk for chromosome abnormalities that would otherwise be false negatives by NIPT; others suggest that NT measurement alone does not add benefit in detecting aneuploidy when NIPT has been performed [7–9]. We undertook this study to assess the performance of NIPT in cases with different cutoffs of NT, which can be helpful for prenatal counselling.

## Methods

### Participant recruitment

We performed a retrospective cohort study of pregnancies who underwent NIPT with NT ≥ 2.5 mm between January 2016 and December 2021. Twin pregnancies were excluded. Demographic characteristics, NT thickness, NIPT results, chromosomal diagnostic findings and pregnancy outcomes were recorded. Consultation with a genetic counselor or clinical geneticist was necessary before performing prenatal diagnosis. This study was approved by the Hospital Ethics Committee and informed consent was obtained from each participant.

### NIPT

For NIPT, 5 mL maternal blood sample was withdrawn and cell-free DNA was extracted from 200 mL plasma using the Extraction and Purification Kit for Human Peripheral Blood Genomic DNA (BGI-Wuhan, Wuhan, People’s Republic of China). Detection Kit for Noninvasive Fetal Trisomy (BGIWuhan) was used for library construction. The pooled library was sequenced by BGISEQ-500 sequencer (BGI-Wuhan) according to the manufacturer’s instruction [10]. Whole-genome shallow massively parallel sequencing was performed in all cases at a depth of about 0.1 time. The z-score cutoff was set at 3 for calling trisomies. Aberrations detectable by genome-wide NIPT include autosomal trisomies of chromosomes 1–22, segmental chromosomal aneuploidy > 10Mb size of chromosomes 1–22 and ± sex chromosome aneuploidy (SCA).

### NT

NT thickness was measured at the gestational age between 11^+0^ and 13^+6^ weeks (CRL 45-84 mm). Fetuses were examined via transabdominal ultrasonography using high-resolution ultrasound machines (Acuson Sequoia 512, Antares, and S2000; SIEMENS Medical Solutions, Mountain View, CA, USA) with 4.0–6.0 MHz curvilinear transducers. NT thickness was measured according to the guidelines of The Fetal Medicine Foundation, London [11] by certified physicians. In our hospital, NT ≥ 2.5 mm is the definition of an increased NT.

### Chromosomal diagnostic testing

For pregnancies and infants who chose chromosomal diagnostic testing, karyotyping analysis and chromosome microarray analysis (CMA) were performed. Briefly, karyotyping was processed using a conventional G-banding method and CMA was performed by CytoScan 750 K array (Affymetrix, USA) according to the manufacturer’s instructions. Written informed consent was obtained from pregnancies who underwent invasive prenatal diagnosis. After a normal CMA in the presence of abnormal clinical symptoms or ultrasound, genetic counseling may turn to the option of testing for single gene disorders. In this condition, exome or whole genome sequencing can be offered.

### Statistical analysis

Positive predictive value (PPV) was calculated as the number of cases for which NIPT screening and confirmatory diagnostic testing were concordant (including mosaic karyotype), divided by the number of cases with diagnostic results, multiplied by 100. Fisher's precision probability test was applied to analyze the statistical significance of PPV in different NT cutoffs, using SPSS 20.0 software (SPSS Inc., Chicago, USA). *P* < 0.05 was considered statistically significant.

## Results

### General findings

During the study period, 1470 singleton pregnancies who with NT ≥ 2.5 mm and underwent NIPT were enrolled in our cohort. Group A (NT 2.5–2.9 mm) included 864 fetuses, Group B (NT 3.0–3.4 mm) included 350 fetuses, and Group C (NT ≥ 3.5 mm) included 256 fetuses. The demographics of the cohort are displayed in Table 1.

**Table 1:** Demographic characteristics of cohort

	Group A (NT 2.5–2.9 mm)	Group B (NT 3.0–3.4 mm)	Group C (NT ≥ 3.5 mm)
Pregnancies number	864	350	256
Maternal age (years)	30.9 ± 4.5	31.0 ± 4.4	32.1 ± 5.0
Gestational age (weeks) for NIPT	14.5 ± 2.6	13.8 ± 2.3	13.4 ± 2.0
Chromosome diagnostic testing	74 (8.4%)	95 (27.1%)	141 (55.1%)

*NIPT*, non-invasive prenatal testing

### Abnormal NIPT results

**Fig. 1:**
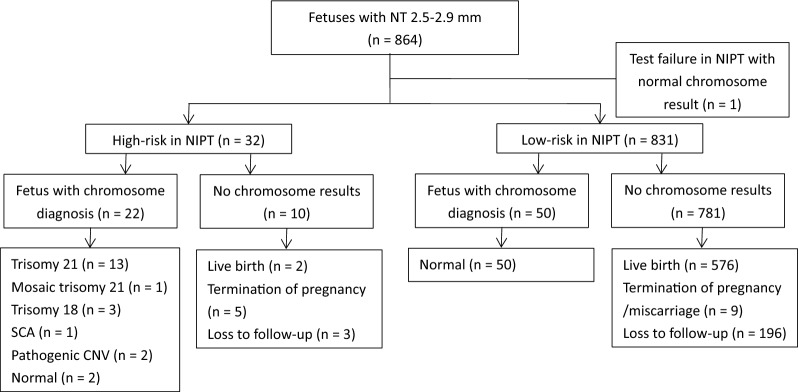
Flowchart of 864 fetuses with NT 2.5–2.9mm. SCA, sex chromosome aneuploidies

**Fig. 2:**
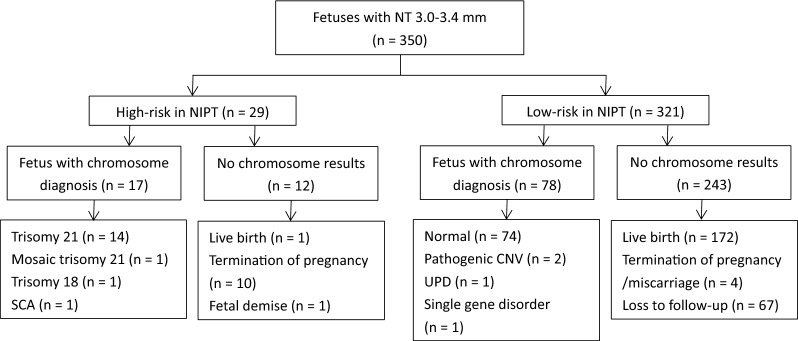
Flowchart of 350 fetuses with NT 3.0–3.4 mm. SCA, sex chromosome aneuploidies; UPD, uniparental disomy

**Fig. 3:**
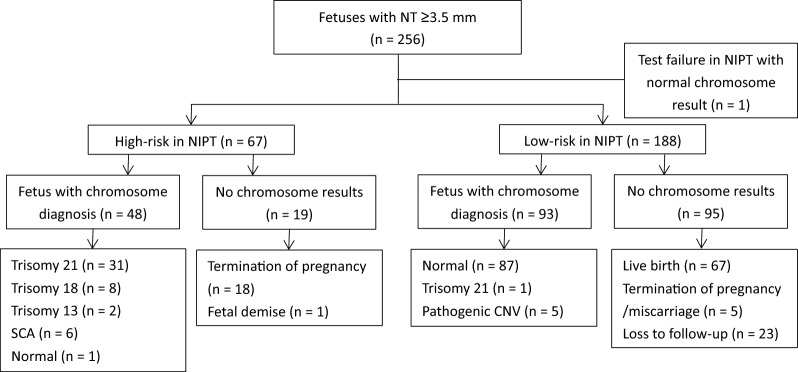
Flowchart of 256 fetuses with NT ≥ 3.5 mm. SCA, sex chromosome aneuploidies

There were 32, 29 and 67 pregnancies received high-risk results in NIPT in Group A, B and C respectively. As showed in Table 2, chromosome results were available in 22/32 (68.8%) in Group A, 17/29 (58.6%) in Group B and 48/67 (71.6%) in Group C. Their chromosomal findings according to the group are summarized in Figs. 1, 2 and 3. The PPV for NIPT was 90.9% (95% CI: 77.9%-100%), 100% and 97.9% (95% CI: 93.7%-100%) in Group A, B and C respectively. Non-significant differences were found in the PPV of different cutoffs of NT (*P* = 0.253). False positive cases were found two in Group A and one in Group C. All of the three false positive cases were positive screens for sex chromosome aneuploidies (SCA) in NIPT, including two cases of 45, X and one case with 47,XYY. All of them finally resulted in live-born healthy infants. Among high-risk cases, 10 in Group A, 12 in Group B and 19 in Group C declined invasive prenatal testing. All of high-risk cases without chromosome results in Group C chose to terminate or suffer from fetal demise. Among them, 55.6% (10/18) of termination of pregnancies had NT ≥ 3.5 mm with structural abnormalities in fetuses. Figures 1, 2 and 3 outlines the overall outcome for high-risk pregnancies who declined prenatal diagnosis.

**Table 2:** Performance of NIPT in screening chromosomal aberrations in different nuchal translucency thickness groups

	Group A (NT 2.5–2.9 mm) n = 864	Group B (NT 3.0–3.4 mm) n = 350	Group C (NT ≥ 3.5 mm) n = 256
High-risk in NIPT	32 (3.7%)	29 (8.3%)	67 (26.2%)
Chromosome result available	22 (68.8%)	17 (58.6%)	48 (71.6%)
Chromosomal aberrations	20	17	47
No aberrations detected	2	0	1
Positive predictive value	90.9%	100%	97.9%

### Normal NIPT results

For cases with low-risk in NIPT, 50 out of 831 in Group A, 78 out of 321 in Group B and 93 out of 188 in Group C underwent chromosome diagnosis, giving a diagnostic rate 6.0%, 24.3% and 49.5% respectively. According to the chromosome diagnosis results, all cases in Group A were true negative. On the total of 78 cases in Group B, prenatal or postnatal diagnostic testing demonstrated chromosomal aberrations in 4 fetuses, suggesting a residual risk 1:20 (5%, 95%CI: 0.1–10.1%). Specifically, these four chromosomal aberrations were two cases of pathogenic CNV, one case of uniparental disomy and one case of single gene disorder. As for Group C, six false negatives were found, including one case of trisomy 21 and five cases of pathogenic CNV. The residual risk for a chromosomal aberration was calculated to be 1:15 (6.5%, 95%CI: 1.4–11.5%) in Group C. The details of these false negative cases are provided in Table 3.

**Table 3:** Overview of false negative cases in NIPT. cFTS, combined first-trimester screening

Case	Maternal age (y)	NT (mm)	Other chromosome diagnosis indication	Chromosome diagnosis time	Chromosome diagnosis result
1	26	3.0	Seizures appeared 20 days after birth	Postnatal	CDKL5 c.1136_1139 del TG
2	34	3.0	None	Prenatal	arr[GRCh37] 14q11.2q31.2 × 2 hmz, 63.815Mb, arr[GRCh37] 14q31.2q32.33 × 2 htz, 22.939Mb
3	30	3.2	High-risk in cFTS	Prenatal	arr[GRCh37]16p11.2 × 1, 597kb
4	33	3.3	High-risk in Cfts Abnormal ultrasound finding (cystic hygroma; nasal bone dysplasia)	Prenatal	arr[GRCh37] 9q34.3 × 1, 332Kb
5	33	3.7	High-risk in cFTS	Prenatal	arr[GRCh37] 16p11.2 × 1, 761 Kb
6	26	3.8	Abnormal developmental assessment at 18 months	Postnatal	arr[GRCh37] 15q11.2q13.1 × 1, 6.22Mb
7	33	4.6	High-risk in cFTS	Prenatal	47,XN, + 21
8	30	4.9	Abnormal ultrasound finding (aberrant left subclavian artery, dextro-aortic arch)	Prenatal	arr[GRCh37]22q11.21 × 1, 3.152Mb
9	27	5.2	High-risk in cFTS	Prenatal	arr[GRCh37]Xp21.1 × 2, 628kb, including Duchenne muscular dystrophy gene
10	30	5.9	Abnormal ultrasound finding (right clubfeet)	Prenatal	arr[GRCh37]9q21.33q31.2 × 1, 20.376 Mb

## Discussion

This study assessed the NIPT performance in cases with different cutoffs of NT. We described the NT, NIPT and chromosomal diagnostic results in a cohort of 1470 single pregnancies from January 2016 to December 2021 at a single medical center. Our study evaluated the PPV of NIPT and found no significant difference in different groups, which demonstrated that the performance of NIPT for screening chromosomal aberrations were similar in cases with NT 2.5–2.9 mm, NT 3.0–3.4 mm and NT ≥ 3.5 mm. It was worth mentioning that in our cohort, there was a case with NT of 4.3mm, screened for 47,XYY in NIPT, finally resulted normal in fetal karyotype and CMA, indicating that false positive still exist in case with abnormal results both in NT and NIPT.

With the present state of technology, NIPT should be able to detect common whole-chromosome aneuploidies with high sensitivity and specificity [3]. Several studies revealed that a NT measurement alone does not add benefit in detecting aneuploidy when cfDNA screening has been performed [9, 12, 13]. Conversely, our data showed one false negative of trisomy 21 with a NT of 4.6 mm. Moreover, the other nine false negative cases including pathogenic CNV, uniparental disomy and single gene disorder were found in fetuses with NT ≥ 3.0 mm. Thus, we confirmed that pregnancies who screen normal in NIPT remain at risk for chromosomal aberrations, especially pathogenic CNV and even common trisomies, which stood in accordance with Kelley et al.’s review [7]. It was reported that with low-risk in NIPT, the frequency of clinically significant condition being detected after prenatal diagnosis is 3.5–6.1% in fetuses with NT ≥ 3.5 mm and lower at 1.5–1.9% in fetuses with NT 3.0–3.4 mm [7, 8]. Base on the presented data, the residual risk after normal NIPT results is 1:20 in fetuses with NT 3.0–3.4 mm and 1:15 in fetuses with NT ≥ 3.5 mm. Our data confirmed the previously published data and showed that NT measurement has an additional value in assessing the residual risk for chromosomal aberrations in the individual fetus with normal NIPT [14–17]. Therefore, we fully agree that invasive diagnosis should provided to fetus with NT ≥ 3.0 mm. Additionally, increased NT has been associated with a higher risk for congenital heart defects, and can provide valuable information about which pregnancies should be offered fetal echo in the second trimester [18].

When focus on NT 3.0–3.4 mm, Petersen et al.’s study supported offering invasive testing as an appropriate choice instead of NIPT to make early diagnosis of clinically important chromosomal aberrations [14]. According to guidelines in China, NIPT should only be used in pregnancies with gestational age more than 12 weeks. Although the guideline recommends that NIPT should carefully be used in pregnancies with ultrasound abnormalities, considering NIPT was incorporated into the public health program by Shenzhen governmment since 2017 and NIPT is free of charge for pregnant women with maternity insurance, a significant proportion of pregnancies with increased NT hoped that the negative NIPT result would reduce their anxiety and stress before they underwent invasive procedure and received amniocentesis diagnostic results. In addition, chorionic villus sampling is not commonly offered to pregnancies with increased NT or positive NIPT considering the risk of confined placental mosaicism. A NIPT before amniocentesis diagnostic would give pregnancies early information of their fetuses’ risk of having common trisomy. In our opinion, when NIPT is performed for free and consultation is comprehensively, it’s acceptable to offer NIPT to pregnancies with increased NT before an invasive procedure.

After low-risk NIPT, we found two pathogenic CNV and one uniparental disomy in fetuses with NT 3.0–3.4 mm and five pathogenic CNV in fetuses with NT ≥ 3.5 mm. CMA has higher resolution than conventional karyotyping, allowing the detection of smaller, submicroscopic imbalances, even some cases of uniparental disomy (i.e., involving isodisomy), by SNP array [19]. Therefore, our data also suggested that CMA may have an additional value in chromosome diagnosis not only in fetuses with NT ≥ 3.5 mm, but in fetuses with NT 3.0–3.4 mm as well, which was agreed with previous studies [14, 20–23]. Except routine screening for aneuploids, any pregnancy whose goal is to maximize the diagnostic yield of chromosome aberrations in their pregnancy should be offered prenatal diagnosis with microarray.

We would like to point out three possible drawbacks to this study. Firstly, the true rate of chromosomal aberrations in this cohort may be underestimated for the reason that some pregnancies opted to terminate without diagnostic testing upon consultation and some diagnoses were made at a later time. Besides, PPV could be impacted by the characteristics of the patients who decided to move forward with diagnostic testing, for example, if the population that elected to have diagnostic testing was enriched for fetuses with additional ultrasound findings (not just an isolated NT), the PPV may be higher because a priori risk for chromosome abnormalities in the population is likely to be higher. Secondly, we had incomplete pregnancy outcomes and therefore cannot determine how many pregnancies loss without diagnostic testing nor how many healthy infants were born. Thirdly, pregnancies with NTs < 2.5 mm were not included in this study, so we can not paint the full picture of how NT and NIPT can be used in practice.

## Conclusion

In conclusion, our findings demonstrated that the PPV of NIPT for screening chromosomal aberrations were similarly in cases with different NT cutoffs, while false positive cases still exist. In this study, we confirmed that pregnancies who screen negative in NIPT with increased NT (≥ 3.0 mm) remain risk for chromosomal aberrations, even common trisomies. Therefore, we suggested offering invasive testing and applying CMA in pregnancies with NT ≥ 3.0 mm, in spite of low-risk in NIPT, to improve the diagnostic yield of chromosomal aberrations for fetuses.

## Data Availability

All data generated or analyzed during this study are included in this published article.
